# Proliferative and granulomatous ileitis in a pig naturally infected with *Lawsonia intracellularis*

**DOI:** 10.1186/s40813-026-00528-y

**Published:** 2026-06-16

**Authors:** Dávid Géza Horváth, Sonja Jeckel, Joaquim Segalés, Bernat Martí-Garcia

**Affiliations:** 1 Department of Pathobiology and Population Sciences, Royal Veterinary College, Hawkshead Lane, North Mymms, Hatfield, Hertfordshire, AL9 7TA UK; 2 Departament de Sanitat i Anatomia Animals, Facultat de Veterinària, Universitat Autònoma de Barcelona (UAB), Bellaterra, Barcelona, Spain; 3 WOAH Collaborating Centre for the Research and Control of Emerging and Re-Emerging Swine Diseases in Europe (IRTA-CReSA), Bellaterra, Barcelona, Spain; 4 Unitat Mixta d’Investigació IRTA-UAB en Sanitat Animal, Centre de Recerca en Sanitat Animal (CReSA), Campus de la UAB, Bellaterra, Barcelona, Spain

**Keywords:** Proliferative enteropathy, Granulomatous enteritis, *Lawsonia intracellularis*, Porcine circovirus 2

## Abstract

**Background:**

Granulomatous enteritis in pigs is mostly associated with porcine circovirus 2 systemic disease (PCV2-SD) but less often caused by fungi, *Salmonella* spp., foreign bodies, *Mycobacterium* spp. or *Lawsonia intracellularis*. This unusual and rare presentation of *Lawsonia intracellularis* infection can only be diagnosed by histopathological and immunohistochemical confirmation.

**Case presentation:**

Postmortem examination was performed in three 9-10-week-old post-weaned growing pigs from an outdoor farm submitted with a clinical history of increased mortality and wasting over the last two batches. Gross findings in all animals included poor body condition, segmental fibrino-necrotizing enteritis and catarrhal colitis. Histopathological examination identified chronic segmental proliferative enteritis with concurrent granulomatous inflammation of the submucosa and Peyer’s patches in one animal (pig A). *Lawsonia intracellularis* was demonstrated in the apical cytoplasm of proliferating crypt enterocytes and in multinucleated giant cells and macrophages of the lamina propria and submucosa by Warthin–Starry stain and immunohistochemistry. PCV2 antigen was not detected in the intestinal lesions or in other lymphoid tissues by immunohistochemistry. Mycobacteriosis was ruled out by means of Ziehl Neelsen stain. Selective culture of intestinal contents for *Salmonella spp.* was negative and *Brachyspira pilosicoli* was isolated and identified by PCR from two animals (pigs A and C). This spirochete could have contributed to the colitis and wasting in these animals. Porcine reproductive and respiratory syndrome virus involvement was discarded in the three pigs by spleen RT-PCR.

**Conclusion:**

This case report highlights *Lawsonia intracellularis* as a rare but significant differential diagnosis for granulomatous enteritis in pigs and emphasizes the importance of achieving a definitive diagnosis by excluding PCV2-SD and demonstrating co-localization of the bacterium with granulomatous inflammation by Warthin–Starry stain and immunohistochemistry.

## Background

The porcine proliferative enteropathy (PE) complex is an infectious disease of post-weaned pigs with classical clinical and pathological manifestations including porcine intestinal adenomatosis (PIA), necrotic enteritis and proliferative hemorrhagic enteropathy. PE is an endemic, global disease of pigs with significant economic impact and is caused by an obligate intracellular Gram-negative bacterium, *Lawsonia intracellularis*, which mostly affects the grower and finisher population [1–4]. Transmission occurs by the fecal-oral route and disease development depends on undefined interactions with gut bacteria and with environmental and management-related stressors. Clinically, PIA is characterised by anorexia, variably severe diarrhea, weight loss and increased mortality. In subclinical infections there is growth retardation without prominent diarrhea. Similar clinical presentations can be caused by swine dysentery (infection with *Brachyspira hyodysenteriae* and with other strongly hemolytic species like *Brachyspira hampsonii* and *Brachyspira suanatina*), intestinal spirochetosis (*Brachyspira pilosicoli* infection), intestinal salmonellosis (for example *Salmonella enterica* Typhimurium infection), post-weaning colibacillosis *(Escherichia coli* infection) and porcine circovirus 2 systemic disease (PCV2-SD). The characteristic pathological feature of PIA is the thickening of the intestinal mucosa, particularly of the distal small intestine, due to immature crypt epithelial cell proliferation. In necrotic enteritis, extensive coagulative mucosal necrosis and fibrin exudation result in the formation of a pseudomembrane (formerly termed as diphtheric membrane). In hemorrhagic enteropathy there is extensive hemorrhage into the lumen presumably due to widespread mucosal diapedesis. Hemorrhagic enteropathy, also known as acute PE, can be associated with sudden death. Lesions mostly occur in the ileum, but the jejunum, cecum and colon can also be affected [1–5]. *Lawsonia intracellularis* can be demonstrated using the Warthin-Starry stain, but immunohistochemistry (IHC) and in situ hybridisation (ISH) are the gold standards for postmortem PE diagnosis as silver impregnation methods can highlight spirochetes as well [2, 5].

## Case presentation

**Fig. 1:**
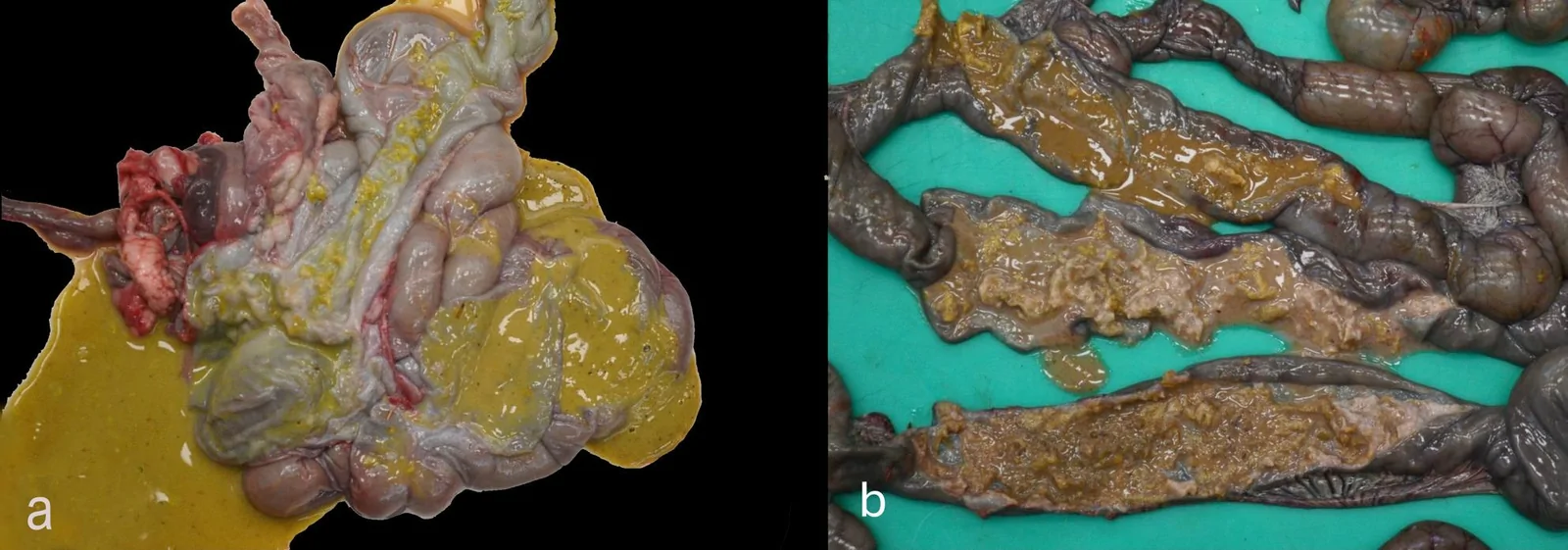
**a**: Large intestine; Pig (A) The cecum contains a moderate amount of fibrin. The spiral colon contains a moderate amount of watery ochraceous content. **b**: Small intestine; Pig (B) The distal small intestine is filled by abundant fibrin admixed with light, brown liquid digesta

Three untreated 9-10-week-old, commercial crossbred pigs (A, B and C) originating from a 120-sow, farrow-to-finish farm in the United Kingdom were submitted for diagnostic postmortem examination to the RVC-FAPD (Royal Veterinary College—Farm Animal Pathology and Diagnostic Services), the local surveillance pathology partner postmortem provider for the Animal and Plant Health Agency (APHA) after being found dead. The farm experienced increased mortality and wasting of pigs in the last two batches starting approximately five weeks post-weaning (~ 9 weeks of age). Mortality of 15 pigs was reported in the most recent affected batch of 100 over one week. Piglets were vaccinated against PCV2 at weaning (3–4 weeks of age). Upon postmortem examination, all carcasses were in poor body condition with generalised marked skeletal muscle atrophy and low body weights (5.5–7 kg). The small and large intestines were flaccid with a moderate amount of yellow to green fluid digesta. This intestinal content was admixed with a moderate amount of friable, yellow, semisolid material (fibrin) in the distal ileum, cecum and proximal colon in pigs A and C, and in the distal small intestine and colon in pig B (Fig. 1). The wall of the distal ileum was moderately thickened in pig A, and subjectively mildly thickened in pigs B and C. Advanced autolysis of pigs B and C precluded any further meaningful macroscopic interpretations. In the distal small intestine of pig C there were semisolid cores composed of fibrinous material admixed with necrotic debris and digesta, and a small amount of partially undigested feed in the colon. None of the pigs had formed feces in the rectum. Segmental fibrino-necrotizing enteritis and catarrhal colitis were diagnosed in all three piglets based on gross examination. Perimortem or incidental findings included pulmonary congestion in all pigs, marked leptomeningeal congestion and multifocal cortical renal cysts in pig A, and mild catarrhal tracheitis in pigs B and C. Pig B was markedly autolysed, limiting subsequent additional testing.

Sampling for ancillary testing from the most representative and least autolysed pig was performed. Bacterial swabs were collected from the liver, lung and spleen from pig A and submitted for general bacteriology; large intestinal contents from pigs A and C were submitted for *Salmonella* spp. and *Brachyspira* spp. cultures as well as *Brachyspira* spp. polymerase chain reaction (PCR). Spleen from all three animals underwent RT-PCR for porcine reproductive and respiratory syndrome virus (PRRSV) and tested negative. *Brachyspira pilosicoli* was detected by PCR from the intestinal contents of pigs A and C (with Ct values of 27.12 and 32.25) and isolated from the intestinal content of pig A. No bacteria were isolated after 48 h of culture from the systemic swabs (liver, lung and spleen) of pig A. *Salmonella* spp. was not cultured from the intestinal contents of pig A and C (Table 1).

**Table 1:** Ancillary tests and results from the three pigs

	Pig A	Pig B	Pig C
Routine culture	No growth	Not performed	Not performed
*Salmonella* culture	No growth	Not performed	No growth
*Brachyspira* culture	BP isolated	Not performed	Not performed
*Brachyspira* spp. PCR	BP detected (Ct 27.12)	Not performed	BP detected (Ct 32.25)
PRRSV RT-PCR	Negative	Negative	Negative

Piglet B was markedly autolysed and considered suboptimal for further testing

BP = *Brachyspira pilosicoli*

**Fig. 2:**
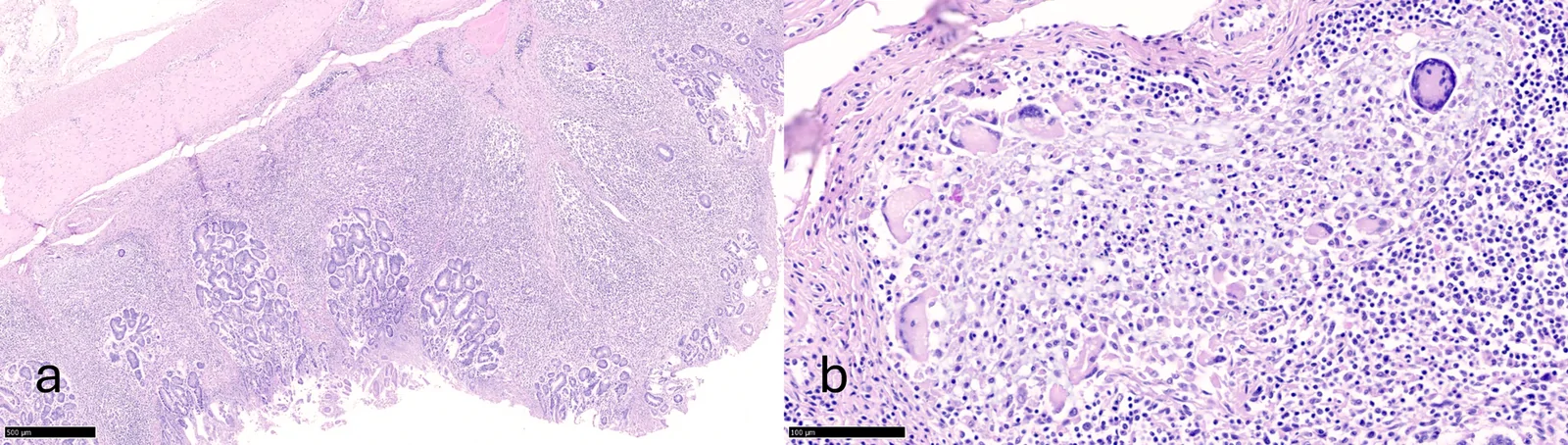
**a**: Ileum; pig A. The intestinal mucosa is moderately to markedly expanded by branching and tortuous crypts composed of hypertrophic and hyperplastic epithelium. The submucosa is markedly thickened by a lymphoplasmacytic infiltrate and mature fibrous connective tissue. HE. Bar = 500 μm. **b**: Ileum; pig A. In the follicles of the Peyer’s patches there is a multifocal, moderate histiocytic infiltrate containing abundant multinucleated giant cells and a few neutrophils. HE. Bar = 100 μm

Multiple organ samples, including lymph nodes, tonsil, lung, spleen and intestinal tract were collected from all pigs, fixed by immersion in 4% buffered formalin for 24 h, routinely processed for histopathology and stained with haematoxylin and eosin. In addition, cerebellum, kidney and liver from pig A, cerebellum from pig B and kidney from pig C were routinely processed for histopathology. The intestinal samples of pig A were stained with Warthin-Starry, Periodic-acid Schiff (PAS) and Ziehl Neelsen following the observation of unusual ileal lesions by haematoxylin and eosin staining. Immunohistochemistry (IHC) for PCV2 in lymphoid organs, lungs and intestines as well as IHC for *Lawsonia intracellularis* were performed in this pig. Microscopically the intestinal mucosa of the small intestine was segmentally expanded by an abundant crypt density in pig A. The crypts showed extensive branching and were tortuous, extending deep into the submucosa and lined by hypertrophic and hyperplastic epithelium containing decreased numbers of goblet cells (Fig. 2a). These abnormal crypts were surrounded by mild neutrophilic, histiocytic and lymphoplasmacytic infiltrates. Crypts were multifocally filled with degenerate neutrophils and karyorrhectic debris (crypt abscesses). The submucosa was segmentally markedly thickened by a lymphoplasmacytic infiltrate and fibroplasia. The gut associated lymphoid tissue (GALT) frequently contained proliferating crypts. In the follicles of the Peyer’s patches there was a multifocal moderate histiocytic infiltrate containing abundant multinucleated giant cells and few neutrophils (Fig. 2b). In the spleen, tonsil, superficial inguinal lymph node and mesenteric lymph node there was diffuse mild loss of follicular definition with mild to moderate reduction in lymphoid cell density (lymphocyte depletion). The organs of pigs B and C showed advanced autolysis and were sub-optimally preserved for detailed histopathological examination. However, within these limits, the most significant histological finding was a thick eosinophilic pseudo-membrane composed of cellular debris, feed particles and numerous bacterial colonies enmeshed in fibrin strands overlying the distal small intestine of these two pigs. The intestinal mucosa was subjectively overcrowded and multifocally minimally extended into the superficial submucosa, however, advanced autolysis precluded any further meaningful interpretation. The colonic lamina propria was infiltrated by a mild lymphoplasmacytic infiltrate in pig C, mild lymphoid depletion was observed in pig B and lymphoid hyperplasia of the submandibular lymph node was diagnosed in pig C.

**Fig. 3:**
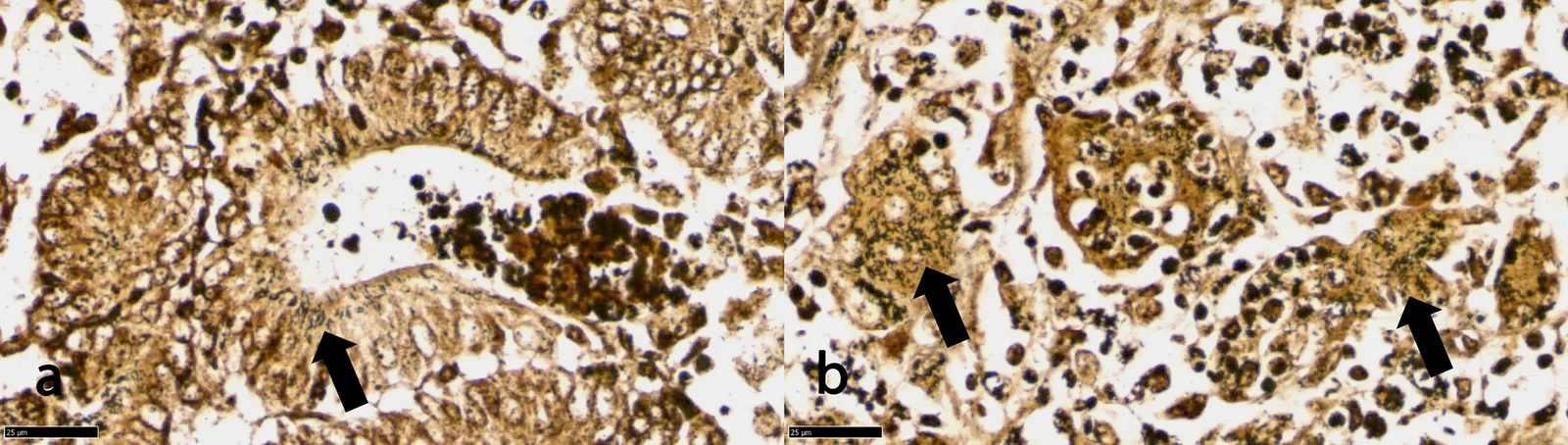
**a**: Ileum; pig A. Numerous small, curved rods (reminiscent of *Lawsonia intracellularis*) are present in the apical cytoplasm of the proliferating crypt epithelium (arrow). Warthin–Starry stain. Bar = 25 μm. **b**: Ileum; pig A. Numerous small, curved rods (reminiscent of *Lawsonia intracellularis*) are present in the cytoplasm of the multinucleated giant cells (arrows). Warthin–Starry stain. Bar = 25 μm

**Fig. 4:**
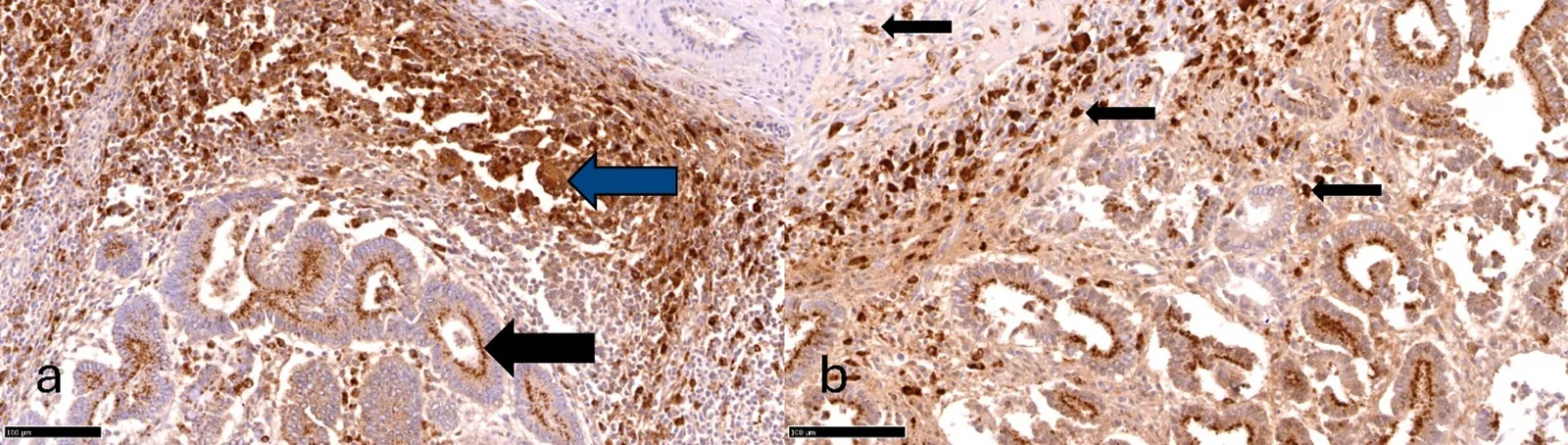
**a** and **b**: Ileum; pig A. *Lawsonia intracellularis* antigen is diffusely present in the apical cytoplasm of proliferating crypt enterocytes (thick black arrow), in the multinucleated giant cells (thick blue arrow), and frequently in the macrophages of the lamina propria and submucosa (thin black arrows). *Lawsonia intracellularis* IHC. Bar = 100 μm

Numerous small, curved rods (reminiscent of *Lawsonia intracellularis*) were identified in the apical cytoplasm of proliferating crypt epithelium and in the cytoplasm of multinucleated giant cells by Warthin–Starry stain in pig A (Fig. 3). Fungal structures or acid-fast organisms could not be demonstrated in the examined sections by PAS and Ziehl-Neelsen stains, excluding mycosis and mycobacteriosis associated with the granulomatous infiltrates. PCV2 antigen was absent in the ileal granulomatous infiltrate or in other lymphoid tissues of pig A. Non-suppurative periarteritis, the hallmark lesion of PCV3-associated disease, was not found in any of the examined pigs. *Lawsonia intracellularis* antigen was detected by IHC diffusely in the apical cytoplasm of proliferating crypt enterocytes (Fig. 4a; black arrow), in the multinucleated giant cells (Fig. 4a; blue arrow), and frequently in the macrophages of the lamina propria and submucosa (Fig. 4b; arrows). The main pathologic findings of the intestinal tract of the three piglets and the special histochemical and IHC results of piglet A are summarised in Table 2.

**Table 2:** Significant post-mortem findings of the intestinal tract of the three pigs, including special stain and IHC results from pig A

	Pig A	Pig B	Pig C
Gross findings	- Segmental fibrino-necrotizing enteritis and catarrhal colitis	- Segmental fibrino-necrotizing enteritis and catarrhal colitis	- Segmental fibrino-necrotizing enteritis and catarrhal colitis
Histologic findings	- Chronic, segmental proliferative enteritis - Chronic, segmental granulomatous enteritis targeting the submucosal lymphoid tissue	- Segmental fibrinonecrotizing enterocolitis	- Segmental fibrinonecrotizing enterocolitis
Warthin–Starry stain	Numerous small, curved rods in the proliferating crypt epithelium and in multinucleated giant cells	Not performed	Not performed
PAS and ZN stain	No agent identified	Not performed	Not performed
PCV2 IHC	No antigen identified	Not performed	Not performed
*L. intracellularis* IHC	Antigen was diffusely detected in the proliferating crypt enterocytes, in the multinucleated giant cells, and in the macrophages of the lamina propria and submucosa	Not performed	Not performed

## Discussion and conclusions

Enteric diseases are a major cause of economic loss, health and welfare issues in the modern pig industry. One of the most significant enteric diseases of swine is PE, which has well defined diagnostic criteria, yet unusual or rare presentations can cause diagnostic challenges even to experienced veterinary professionals.

The significant mortality in this herd and the grossly identified distal fibrino-necrotizing enteritis with catarrhal colitis initially raised suspicion of salmonellosis as the most likely differential. PE was considered less likely due to the lack of evident ileal proliferation and expansion, although a moderate ileal thickening was present in pig A. Characteristic histological changes of PE were identified in pig A which included the thickening of the intestinal mucosa due to immature crypt epithelial cell proliferation. Crypts were elongated, enlarged, and crowded with concurrent marked reduction in goblet cells [2, 4]. Definitive diagnosis required the identification of small, curved bacteria in the apical cytoplasm of proliferative crypt epithelial cells. A classic histopathological technique for their visualisation is the Warthin-Starry stain, but immunohistochemistry is considered the gold standard for postmortem PE diagnosis [2] and both techniques were applied accordingly. An unusual granulomatous enteritis with co-localization of *L. intracellularis* by means of Warthin Starry and IHC in pig A was also observed, which effaced the submucosal lymphoid tissue of the ileum. PCV2 was the most likely etiology associated with this lesion but was excluded by means of IHC. Granulomatous inflammation of gut-associated lymphoid tissue (GALT) is characteristic but not exclusive of PCV2 infection and ancillary testing aimed at determining PCV2 antigen within lesions, like IHC, is required to prove causality [6].

The presence of *Lawsonia intracellularis* antigens within multinucleated giant cells and macrophages of the lamina propria and submucosa suggests a causative role behind this granulomatous response, therefore it was diagnosed as the causative agent for both, the proliferative and granulomatous infiltrate in pig A. PAS, Ziehl Neelsen and PCV-2 IHC were performed but there was no evidence for other agents that could be responsible for such inflammatory reaction (fungi, *Salmonella*, foreign bodies, *Mycobacterium* spp., PCV-2). The isolated *Brachyspira pilosicoli* was likely contributory to the catarrhal colitis and wasting. The target organs for this organism are the cecum and colon, and granulomatous inflammation has not been associated with it. *Brachyspira pilosicoli* can generate a “false brush border” over the surface of colonic enterocytes. Some of these organisms could have been highlighted by our Warthin-Starry stain, but their extracellular location and colonic habitat differentiate them from *Lawsonia intracellularis*. Positivity of the IHC for *L. intracellularis* further supports this organism as the cause for the distal enteritis [4, 7, 8].

*Lawsonia intracellularis* disrupts epithelial cell maturation during intracytoplasmic replication, driving the characteristic adenomatous proliferation. Bacterial antigens are taken up by dendritic cells and macrophages and presented to T cells in local lymphoid tissues, initiating a type 1 T helper (Th1) immune response. This response is characterised by interferon-γ (IFN-γ) production and the activation of cytotoxic T cells and macrophages. *L. intracellularis* is capable of persisting and replicating at low levels within macrophages [3]. The presence of granulomatous inflammation with multinucleated giant cells in PE is a rare but reported phenomenon and highlights the importance of differentiating this condition from PCV2-SD and other causes of granulomatous enteritis in pigs in routine diagnostics [1, 6, 9].

Pigs A and B were diagnosed with segmental fibrino-necrotizing enteritis with catarrhal colitis and intralesional superficial bacterial colonies. The similar gross presentation makes *L. intracellularis* and *B. pilosicoli* coinfection a plausible cause for disease in all three piglets, but obvious mucosal proliferation was not present in pigs B and C, although autolytic changes could have hindered its identification. Granulomatous inflammation was also absent in these animals. It is unknown if the *L. intracellularis* and *B. pilosicoli* coinfection was solely responsible for the severe clinical manifestation in these pigs as PE is generally not fatal [5]. The most significant immunomodulatory viruses of swine (PCV2 and PRRSV) were excluded but other management and/or environment-related factors could have contributed to disease severity. The mild to moderate lymphoid depletion in pig A and B was likely caused by non-specific chronic disease and wasting but other unknown immunosuppressive causes are also possible.

This case report highlights that PE can rarely be associated with a granulomatous distal enteritis/ileitis causing potential diagnostic challenges and should be differentiated from PCV2-SD and other causes of granulomatous enteritis in swine.

## Data Availability

No datasets were generated or analysed during the current study.

## Abbreviations

IHC: Immunohistochemistry
PCR: Polymerase chain reaction
PCV2: Porcine circovirus type 2
PE: Proliferative enteropathy
PRRSV: Porcine reproductive and respiratory syndrome virus
